# Prevalence of Health-risk Behaviors among Government Schools’ Students in Jordan

**Published:** 2017-12

**Authors:** Ahmad Yahya AL-SAGARAT, Mahmoud Taher AL KALALDEH

**Affiliations:** 1. Dept. of Community and Mental Health Nursing, College of Nursing, Mutah University, Al-Karak, Jordan; 2. Faculty of Nursing, Al-Zaytoonah University of Jordan, Amman, Jordan

**Keywords:** Student, Health risk behavior, Government schools, Jordan

## Abstract

**Background:**

Adolescence is a developmental stage associated with many behavioural fluctuations and health risks behaviours. In this study, various health risk behaviours among Government school students in Jordan were assessed.

**Methods:**

This cross-sectional descriptive study recruited 1256 students from 20 secondary schools all over the country. Students completed the Global School-based Student Health Survey (GSHS, 2009–2012). The study was conducted in the period between Feb 2016 and Aug 2016. Chi-square (x^2^) was used to examine differences among the demographic variables.

**Results:**

Students scored low in eating breakfast, eating fruit, vegetables, and milk products. However, students scored moderately in hand and mouth hygiene. Students showed minimal incidences of physical attack and physical fight. Although suicidal attempts were not significantly reported, complaining from worries, feeling of sadness and hopelessness were moderately scored. The majority of physical activities were reported from walking or riding bicycles. However, three hours per day was the average of time spent on sitting activities. Students scored lowest in school absenteeism and the majority described their classmates as kind and helpful. Parental control on students’ home activities was regarded.

**Conclusion:**

In comparison with 2004 and 2007 statistics, students revealed improvements in physical activity, and reduced physical attacks and injuries. Future researchers are encouraged to discover factors associated with these changes.

## Introduction

Adolescence is a unique developmental stage characterized by rapid physiological, psychological, cognitive, and emotional changes (1). Globally, hundreds of thousands of adolescents suffer yearly from serious injuries, physical abuse, and other unhealthy exposures (2–5). Adolescents from both developed and developing countries engage in a variety of risky behaviours associated with eating habits, mouth and hand hygiene, violence, unintentional injuries, self-protection measures, abuse and harassment, emotions and friendships, smoking, and physical activities (4). In Jordan, where 10.97% of the total population ages between 13–16 yr old, the government has special recognition of these health risk behaviours represented by national health promotion programmes (6,7).

Since 2004, the Global School-based Student Health Survey (GSHS) has been applied in Jordan to monitor the prevalence of health risk behaviours associated with this age category (7). This national survey was conducted for a second time in 2007 by the Jordanian Ministry of Health (8). The major difference found between 2004 and 2007 statistics were; 14.3% of students were at higher risk of being overweight, 14.3% were physically active, and 24.9% used different forms of tobacco (7–9).

Therefore, this study aimed to re-assess the Government school students’ health risk behaviours to enhance the level of awareness of both policymakers and future researchers to identify factors surrounding these changes in adolescents’ health behaviours.

## Methods

### Study design

A descriptive cross-sectional study was conducted to assess health risk behaviors among Government school students in Jordan. The study was conducted in the period between Feb 2016 and Aug 2016.

### Sampling Procedure

Cluster sampling technique started with selecting five governorates of Jordan representing all geographical cantons (two from north, three from middle, and two from south). Secondary schools within these governorates were selected randomly and the target was to include four schools from each governorate divided equally between male and female schools. As a multi-stage random sample, students from each school were selected randomly using a computer-generated random numbers technique and based on specific inclusion criteria that included ages between 13–16 yr old, Arabic native speakers, lived more than one year in the same governorate. Students with chronic illnesses, severe visual or auditory deficit, or psychological disturbances were excluded from the study.

The sample size was estimated based on the determination of a confidence interval (CI) at 95%, and statistical power of 80%, and α of 0.05 (9). Based on these, it was planned to recruit at least 60 students randomly from each school.

### Study Procedures

A pilot study was conducted on 45 students using the same inclusion criteria to ensure clarity of the questionnaire before using it in a wider population. Informed consents that required parents/guardians’ signature were provided. Once returned, students were required to complete the questionnaires independently within one hour. Research assistants were recruited and trained to assist in data collection procedure. However, neither research assistants nor schoolteachers influenced students’ responses or provided any assistance to complete the questionnaire.

### Measures

A self-reported questionnaire was the main method for data collection. The study questionnaire consisted of two main sections as follows:

1. Socio-demographic variables that include age, gender, class, nationality, height, weight, father, and mother education (7 Questions).
2. The modified Arabic version of the GSHS (10). The GSHS was developed by WHO and the Centres for Disease Control and Prevention (CDC) and developed successively in 2009 and 2014. This survey with 77 items targeted students aged 13–15 yr to assess their protective and risky behaviours.

Although the questionnaire examined the majority of health-risk behaviours, items related to alcohol and drug use were excluded from the questionnaire upon the request of the ethical committee.

Thus, the final questionnaire consisted of 57 items presented as follows:

1. Eating habits (12 questions)
2. Mouth and hand hygiene (5 questions)
3. Violence (2 questions)
4. Unintentional injuries (3 questions)
5. Self-protection (2 questions)
6. Abuse (2 questions)
7. Emotions and friendships (9 questions)
8. Smoking (6 questions)
9. Physical activities (5 questions)
10. School experience (6 questions)

The validity and reliability of the Arabic version of the GSHS were assessed by numerous studies conducted in different Arabic countries including Jordan [8], as well as non-Arabic versions such as the Persian version (11).

The study protocol was approved by the Mutah University’s Ethical Committee (No. ECI/2015), and the Institutional Review Board (IRB) in the Ministry of Education, Jordan (No: 3/10/6852).

### Statistical Analysis

Data was entered into IBM SPSS software (ver. 20 (Chicago, IL, USA). Initially, data were screened for missing and outliers treated by replacement with means. Descriptive statistics were used to describe the study demographics using frequencies (n), percentages (%), means, and standard deviation (SD). Chi-square (x^2^) was used to examine differences among demographic variables.

## Results

### Participants’ demographic characteristics

Overall, 1256 students participated in this study from three Jordanian cantons (north, middle, and south). The majority of students were Jordanians, recruited from secondary schools, and aged between 15 and 16 yr old.

The distribution was equal about gender showing no statistical difference (*P*=0.955). The mean of Body Mass Index (BMI) was 22 indicating normal body weights. The majority of fathers and mothers were educated at school level, and university education ranked second in the order (Table 1).

**Table 1:** Participants' demographics characteristics (N=1256)

***Demographic***	***N***	***%***	***Sig.***
Canton			
North	423	33.7	0.779
Middle	426	33.9	
South	407	32.4	
School nature			
Primary	312	24.8	<0.001
Secondary	944	75.2	
Age (yr)			
13	42	3.3	<0.001
14	226	18.0	
15	450	35.8	
16	438	42.8	
Gender			
Male	627	49.9	0.955
Female	629	50.1	
Height, mean, SD	160.26	10.48	
Weight, mean, SD	53.98	11.97	
Nationality			
Jordanian	1187	94.5	<0.001
Others	69	5.5	
Father Education			
Illiterate	24	1.9	<0.001
School level	796	63.4	
University level	436	34.7	
Mother Education			
Illiterate	46	3.7	<0.001
School level	661	52.6	
University level	549	43.7	
Total	1256	100	

### General nutritional behaviours

Regarding eating breakfast, the majority of students (mean:3.74/5.0) revealed they were generally eating breakfast. However, the reasons for not taking breakfast were mainly: lack of time and inability to eat in the early morning. Students scored low in eating fruits and vegetables in which both were below the midpoint level. Similarly, eating milk and milk products was below the midpoint. Despite these unfavorable scores, students scored lower in drinking carbonated soft drinks, eating fast foods, and eating high-fat foods. About half the students indicated they received health education classes about healthy foods in the past years (Table 2).

**Table 2:** General nutrition behaviour (N=1256)

***Item***			***Score***
During the past 30 d, how often did you eat breakfast?, mean (SD)			3.74 (1.36)
^*^1–5			
What is the main reason you do not eat breakfast?, n (%)	I always eat breakfast		364 (29.0%)
	I do not have time for breakfast		429 (34.2%)
	I cannot eat early in the morning		328 (26.1%)
	Food is not prepared at home		55 (4.4%)
	There is not always food in my home		7 (0.6%)
	Some other reason		73 (5.5%)
During the past 30 d, how often did you go hungry because there was not enough food in your home?, mean (SD)			1.47 (0.89)
^*^1–5			
During the past 30 d, how many times per day did you usually eat fruit, E.g. apples, oranges, grapes, or bananas?, mean (SD)			2.92 (1.28)
^*^1–5			
During the past 30 d, how many times per day did you usually eat vegetables, such as tomatoes, cucumbers, sweet peppers, or carrots?, mean (SD)			3.31 (1.51)
^*^1–7			
During the past 30 d, how many times per day did you usually drink carbonated soft drinks, such as Coke, Pepsi etc?, mean (SD)			3.08 (1.72)
^*^1–7			
During the past 7 d, on how many days did you eat at a fast food restaurant, such as McDonald’s, Burger King etc?, mean (SD)			2.22 (1.62)
^*^1–8			
During the past 30 d, how many times per day did you usually drink milk or eat milk products, such as yogurt, cheese, or labneh?, mean (SD)			3.02 (1.42)
^*^1–7			
During the past 30 d, how many times per day did you usually eat foods high in fat, E.g fried meat, or fried potatoes?, mean (SD)			2.60 (1.18)
^*^1–7			
During this school year, were you taught in any of your classes the benefits of healthy eating?, n (%)		Yes	566 (45.1%)
		No	690 (54.9%)

* Scores range

### Hand and mouth hygiene

Results regarding hand and mouth hygiene are presented in Table 3. Students’ responses to all queries were above the midpoint, indicating an intermediate-to-higher compliance to these behaviours. More than 46% of students claimed to receive education of the importance of hand hygiene in the past years (Table 3).

**Table 3:** Hand and mouth hygiene (N=1256)

***Item***		***Score***
During the past 30 d, how many times per day did you usually clean or brush your teeth?, mean (SD)		3.20 (1.41)
^*^1–6		
During the past 30 d, how often did you wash your hands before eating?, mean (SD)		4.43 (0.98)
^*^1–5		
During the past 30 d, how often did you wash your hands after using the toilet or la trine?, mean (SD)		4.89 (0.53)
^*^1–5		
During the past 30 d, how often did you use soap when washing your hands?, mean (SD)		4.56 (0.89)
^*^1–5		
During this school year, were you taught in any of your classes the importance of hand washing?, n (%)	Yes	581 (46.3%)
	No	675 (53.7%)

* Scores range

### Physical attack and personal safety

This part illustrates the frequency of experienced physical attack and injuries. Students reported minimal incidences of being attacked, being involved in a physical fight, and seriously injured over the past year (Table 4).

**Table 4:** Physical attack, serious injuries, and personal safety (N=1256)

***Item***			***Score %***
During the past 12 months, how many times were you physically attacked? mean (SD)			1.50 (1.21)
^*^1–8			
During the past 12 months, how many times were you in a physical fight? mean (SD)			2.18 (1.85)
^*^1–8			
During the past 12 months, how many times were you seriously injured? mean (SD)			1.65 (1.02)
^*^1–8			
During the past 12 months, what was the most serious injury that happened to you? n (%)	I was not seriously injured		816 (65.0)
	I had a broken bone		121 (9.6)
	I had a cut or stab wound		106 (8.4)
	I had a concussion or head injury		28 (2.2)
	I had a gunshot wound		7 (0.6)
	I had a bad burn		38 (3.0)
	I was poisoned		2 (0.2)
	Something else		138 (11.0)
During the past 12 months, what was the major cause of the most serious injury that happened to you? n (%)	I was not seriously injured		804 (64.0)
	I was in a motor vehicle accident		38 (3.0)
	I fell		63 (5.0)
	Something fell on me		120 (9.6)
	I was attacked or by someone		28 (2.2)
	I was in a fire		30 (2.4)
	I inhaled or swallowed something		16 (1.3)
	Something else		157 (12.5)
During the past 30 d, how often did you use a seat belt when riding in a car or other motor vehicle driven by someone else?, mean (SD)			1.79 (1.50)
^*^1–5			
During the past 30 d, how many times has someone stolen or deliberately damaged your property in school?, mean (SD)			1.46 (1.06)
^*^1–8			
During this school year, were you taught in any of your classes how to avoid or prevent motor vehicle accidents? n (%)		Yes	720 (57.3)
		No	536 (42.7)

* Scores range

The most common injuries were bone fracture, cut wound, and head injuries. However, the majority of these incidences occurred as a result of falling down and striking with objects, and few students reported motor vehicle accidents and fire. Although the majority (57.3%) claimed attending classes about preventing motor vehicle accidents, they admitted applying of seat belt when getting ride in a motor infrequently (1.79 out of 5.0) (Table 4).

### Feeling and friendships

Students indicated that they felt lonely rarely. They complained moderately from worries that caused sleep disturbance.

More than 43% of them stated they experienced some feeling of sadness or hopelessness lasting two weeks. However, a few suicidal attempts or planning for suicide was reported. The majority of students did not receive sufficient health education about stress and anger management (Table 5).

**Table 5:** Feeling and friendships (N=1256)

***Item***		***Score***
During the past 12 months, how often have you felt lonely?, mean (SD)		2.18 (1.29)
^*^1–5		
During the past 12 months, how often have you been so worried about something that you could not sleep at night?, mean (SD)		2.51 (1.28)
^*^1–5		
During the past 12 months, did you ever feel so sad or hopeless almost every day for two weeks or more in a row that you stopped doing your usual activities?, n (%)	Yes	549 (43.7)
	No	707 (56.3%)
During the past 12 months, did you ever seriously consider attempting suicide?, n (%)	Yes	216 (17.2)
	No	1040 (82.8)
During the past 12 months, did you make a plan about how you would attempt suicide?, n (%)	Yes	159 (12.7)
	No	1079 (87.3)
During the past 12 months, how many times did you actually attempt suicide?, mean (SD)		1.21 (0.68)
^*^1–5		
How many close friends do you have?, mean (SD)		3.57 (0.83)
^*^1–4		
During this school year, were you taught in any of your classes how to handle stress in healthy ways?, n (%)	Yes	216 (17.2)
	No	1040 (82.8)
During this school year, were you taught in any of your classes how to manage anger?, n (%)	Yes	341 (27.1)
	No	915 (72.9)

* Scores range

### Physical activities

Students indicated they performed an averaged 60-min physical activity per day. However, they scored higher in some activities such as walking and riding bicycle. The average number of physical education classes attended each week was two classes or less. However, the majority of students spent an average of three hours per day on sitting activities such as watching television or using computers. The majority did not attend health education class about the physical activities (62.8%) (Table 6).

**Table 6:** Physical activity (N=1256)

***Item***		***Score***
During the past 7 d, on how many d were you physically active for a total of at least 60 min per day?, mean (SD) ^*^1–8		3.87 (2.44)
During the past 7 d, on how many days did you walk or ride a bicycle to or from school?, mean (SD)		5.38 (2.94)
^*^1–8		
During this school year, on how many days did you go to physical education (PE) class each week?, mean (SD)		2.10 (1.65)
^*^1–5		
How much time do you spend during a typical or usual day sitting and watching television, playing computer games, talking with friends, or doing other sitting activities?, mean (SD)		2.71 (1.54)
^*^1–6		
During this school year, were you taught in any of your classes the benefits of physical activity?, n (%)	Yes	467 (37.2)
	No	789 (62.8)

* Scores range

### Experiences at school and home

Positively, students claimed to have minimal school absenteeism as the average of missed classes was less than two classes in the last month. Regarding students’ relationships with their classmates, the majority indicated that other students were kind and helpful. In relation to parents’ or guardians’ monitoring, students scored higher in achieving approved parental control on their homework, understanding of their problems and worries, and observation on their free-time activities (Table 7).

**Table 7:** Experiences at school and home (N=1256)

***Item***	***Score***
During the past 30 d, on how many days did you miss classes or school without permission?, mean (SD)	1.84 (0.99)
^*^1–5	
During the past 30 d, how often were most of the students in your school kind and helpful?, mean (SD)	3.78 (1.35)
^*^1–5	
During the past 30 d, how often did your parents or guardians check to see if your homework was done?, mean (SD)	3.84 (1.39)
^*^1–5	
During the past 30 d, how often did your parents or guardians understand your problems and worries?, mean (SD)	3.52 (1.45)
^*^1–5	
During the past 30 d, how often did your parents or guardians really know what you were doing with your free time? mean (SD)	3.53 (1.48)
^*^1–5	
During the past 30 d, how often did your parents or guardians go through your things without your approval?, mean (SD)	1.71 (1.26)
^*^1–5	

* Scores range

## Discussion

This study shed light on the prevalence of health risk behaviours associated with school students in Jordan. Different health risks behaviours were prevalent according to the statistics obtained in 2004 and 2007.

### General nutritional behaviours

Globally, unhealthy eating was one of the major factors associated with health risk behaviours psychological distress (12, 13). Adolescents’ body weight unveiled in this study was consistent with 2004 and 2007 showing fewer overweight students (7,8). This study showed that the majority of students (74.8%) claimed to eat breakfast ‘most of the time’. This is consistent with other studies conducted in the region. For instance, 56.4% of Jordanian students aged 12–17 yr old ate breakfast most of the time (14). Likewise, a Bahrainian study showed that 51.7% of adolescents aged between 14 – 19 yr also eat breakfast (15). However, the reasons for not taking breakfast as indicated by the students in this study were mainly due to lack of time, and inability to eat in the early morning. A previous study in Jordan suggested the same reasons (16). Adolescents in Jordan were found to be eating less fruit and vegetables in addition to milk and milk products. A previous study in Jordan in 2015 found that the percentage of adolescents’ eating fruit and vegetables was 6.1%. In contrast, the 13.6% of adolescents ate fruit and 25.2% ate vegetables (15). Students in this study scored lower in drinking carbonated soft drinks and eating fast foods which is consistent with a former study in 2015 (16).

### Hand and mouth hygiene

Students in the current study scored intermediately in brushing teeth. They scored higher in washing hand before eating and after toileting and using soap, which was also consistent with 2004 and 2007 statistics. This significant improvement in tooth brushing behaviour was also remarkable in the European countries (17). This improvement was also reported in the South Asian countries due to increase awareness of the importance of self-hygiene in preventing the transmission of diseases (3).

### Physical attack and personal safety

Generally, serious physical attacks and injuries were at the lowest level among students in this study. These studies were inconsistent with 2004 and 2007 results (7,8). Physical fights were reported by less than 47.0% (7,8). These results were inconsistent with Omani study that showed that 47.6% of students aged 13 to 15 yr were involved in a physical fight, of which 40.9% were seriously injured (18). Likewise, in Qatar (38.3%) (19). the significant reduction in physical fights, serious injuries from 2004 and 2007 compared to 2016 might be related to the improvements in school environments and health promotion programmes (20).

### Feeling and friendships

Students in this study indicated feeling loneliness rarely. This figure was consistent with 2004 and 2007 statistics (15.7% and 15.8%, respectively). Students complained moderately from worries that caused sleep disturbance and more than 43% stated feeling of sadness or hopelessness that interrupted their usual activities. This was consistent with a previous Jordanian study that found that 18.4% expressed worries about something that disturbed their sleep (8). Although less than 18% of students admitted serious suicide attempts or planning for suicide, a growing incidence might be a result of lack of health education and parental monitoring (8).

### Physical activities

About half of the participating students claimed performing an averaged 60-min physical activity per day. Most of these physical activities were walking and riding bicycles. These results indicated an improvement in physical activity among adolescents compared with 2004 and 2007 statistics (7, 8). Adolescents in other countries showed a less adherence to physical activity compared to Jordan such as the Omani students (16.0%) (18), Yemeni students (15.2%) (21), Moroccan students (14.4%) (22), and American students (27.1%) (23). However, Bahraini students showed 41.7% (15). Students in this study claimed to spend an average of three hours per day in sitting activities such as watching television or using computers similar to a previous study conducted in Jordan (16). However, in previous large survey studies conducted in the US and Chinese, students, physical inactivity was not a major factor associated with health risk behaviours (12, 13, 23).

### Experiences at school and home

School absenteeism was intermediately reported by the majority of students that was also consistent with the previous report in 2007 (8). While students in this study revealed that other students were kind and helpful, this figure was inconsistent with previous reports in Jordan (7,8). Parents’ surveillance on students’ home activities were positively regarded and consistent with previous report (8).

### Limitations

Students in this study were recruited from public schools only that may limit the generalizability of findings to all students from other educational sectors in Jordan.

## Conclusion

There are some health risk behaviours among adolescent Jordanian students. There is a need for an effective health education program especially for eating a healthy diet and using proper mouth hygiene. In addition, most of risky behaviours were accompanied with lack of educational classes in the school that may urge educational authorities to develop new policy regarding promoting students’ health. In comparison to previous reports, there is a significant improvement in some of health behaviours such as increasing physical activity, and decreasing physical fight and attacks, and physical injuries. The role of parents in controlling health behaviour in is collaborative with school.

## Ethical considerations

Ethical issues (Including plagiarism, informed consent, misconduct, data fabrication and/or falsification, double publication and/or submission, redundancy, etc.) have been completely observed by the authors.
